# Non-coding RNAs as potential biomarkers in osteosarcoma

**DOI:** 10.3389/fgene.2022.1028477

**Published:** 2022-10-19

**Authors:** Lijuan Fan, Zhenhao Zhong, Yubo Lin, Jitian Li

**Affiliations:** ^1^ Henan Luoyang Orthopedic Hospital (Henan Provincial Orthopedic Hospital), Zhengzhou, Henan, China; ^2^ Luoyang Postgraduate Training Department, Henan University of Chinese Medicine, Zhengzhou, Henan, China; ^3^ Department of Spinal Surgery, The First Affiliated Hospital of Hainan Medical University, Haikou, Hainan, China; ^4^ School of Clinical Medicine, Guilin Medical University, Guilin, Guangxi, China; ^5^ The First College for Clinical Medicine, Guangzhou University of Chinese Medicine, Guangzhou, Guangdong, China

**Keywords:** osteosarcoma, non-coding RNAs, biomarker, diagnosis, treatment, prognosis

## Abstract

Osteosarcoma (OS) is a primary solid malignant tumor that occurs most frequently in the metaphysis of long bones. More likely to happen to children and adolescents. OS has high mortality and disability rate. However, the etiology and pathogenesis of OS have not been fully understood till now. Due to the lack of effective biomarkers, OS cannot be precisely detected in the early stage. With the application of next-generation and high-throughput sequencing, more and more abnormally expressed non-coding RNAs(ncRNAs) have been identified in OS. Growing evidences have suggested the ncRNAs, such as microRNAs (miRNAs), long non-coding RNAs (lncRNAs), circular RNAs (circRNAs), have played an important role in the tumorigenesis and progression of OS. Thus, they can be served as novel biomarkers for diagnosis, treatment and prognosis. This review summarized the application of ncRNA as biomarkers in OS in detail, and discussed the limitation and future improvement of the potential biomarkers.

## Introduction

Osteosarcoma (OS), as a common kind of primary malignant tumor of bone, has a high mortality (Cersosimo et al., 2020), accounting for about 2.4% of children with malignant tumors and 20% of all primary bone cancers. Depending on statistics, there are about 800 new cases in the United States each year, among which about 50% are children and adolescents (Ward et al., 2014). Studies have shown that the incidence of OS in men is higher than that in women, which is 1.27 times that of women. (Lee J. A. et al., 2021). OS occurs in the epiphysis of long bones more frequently, such as the proximal tibia, distal femur, proximal humerus, and other parts of the fastest growing bone (Zhao et al., 2018). OS has a high disability and mortality, and is prone to metastasis (Zheng et al., 2020). The prognosis of patients with early metastasis are very poor, the 5-year survival rate of patients with metastasis is lower than 20% (Thanindratarn et al., 2019). However, the early symptoms of OS are not typical, it was usually in the late stage when found even with metastasis, resulting in the high mortality rate. Early diagnosis and treatment are very important for the prognosis of OS. With the continuous improvement of the diagnosis and treatment methods, a variety of therapies have emerged, including targeted therapy and immunotherapy, which have effectively reduced the overall mortality of the patients. Nevertheless, the prognosis is still far from ideal. Therefore, it is urgent to carry out in-depth research on the related molecular mechanisms of the occurrence and development. By looking for new and effective biomarkers for detection, diagnosis, therapy and prognosis, the disability rate and mortality rate can be reduced in some extent, so as to prolong the survival time of the patients.

There are a wide kinds of tumor markers. At present, the discovered species include nucleic acid, protein, glucose, small molecular metabolites, cytokines, circulating tumor cells (CTCs), and so on, which play an important role in the diagnosis, treatment and prognosis of tumors. In the past few decades, bioinformatics has provided new ideas for the functions of early diagnosis and treatment with its advantages of simplicity, non-invasive and high efficiency, further play an important role in early diagnosis and prognosis evaluation. Compared with protein biomarkers, ncRNAs tumor markers have the advantage of replicability, stable expression, less affected by *in vivo* and *in vitro* stimulation. In clinic, the commonly used ncRNAs tumor markers of OS include microRNA (miRNA), long non-coding RNA (lncRNA), and circular RNA (circRNA) (Inamoto et al., 2018; Yang et al., 2021). Biomarkers have become a new potential due to their advantages of easy sample acquisition, less trauma to the human body, higher sensitivity and specificity. These biomarkers have played significant roles in non-invasive diagnosis and detection during the occurrence, development and prognosis of tumor. Non-coding RNA (ncRNAs), because of its own advantages, has become a major participant in tumor markers, such as in hepatocellular carcinoma, lung cancer, breast cancer, et al. (Wong et al., 2018; Iqbal et al., 2019; Shen et al., 2020).

**FIGURE 1:**
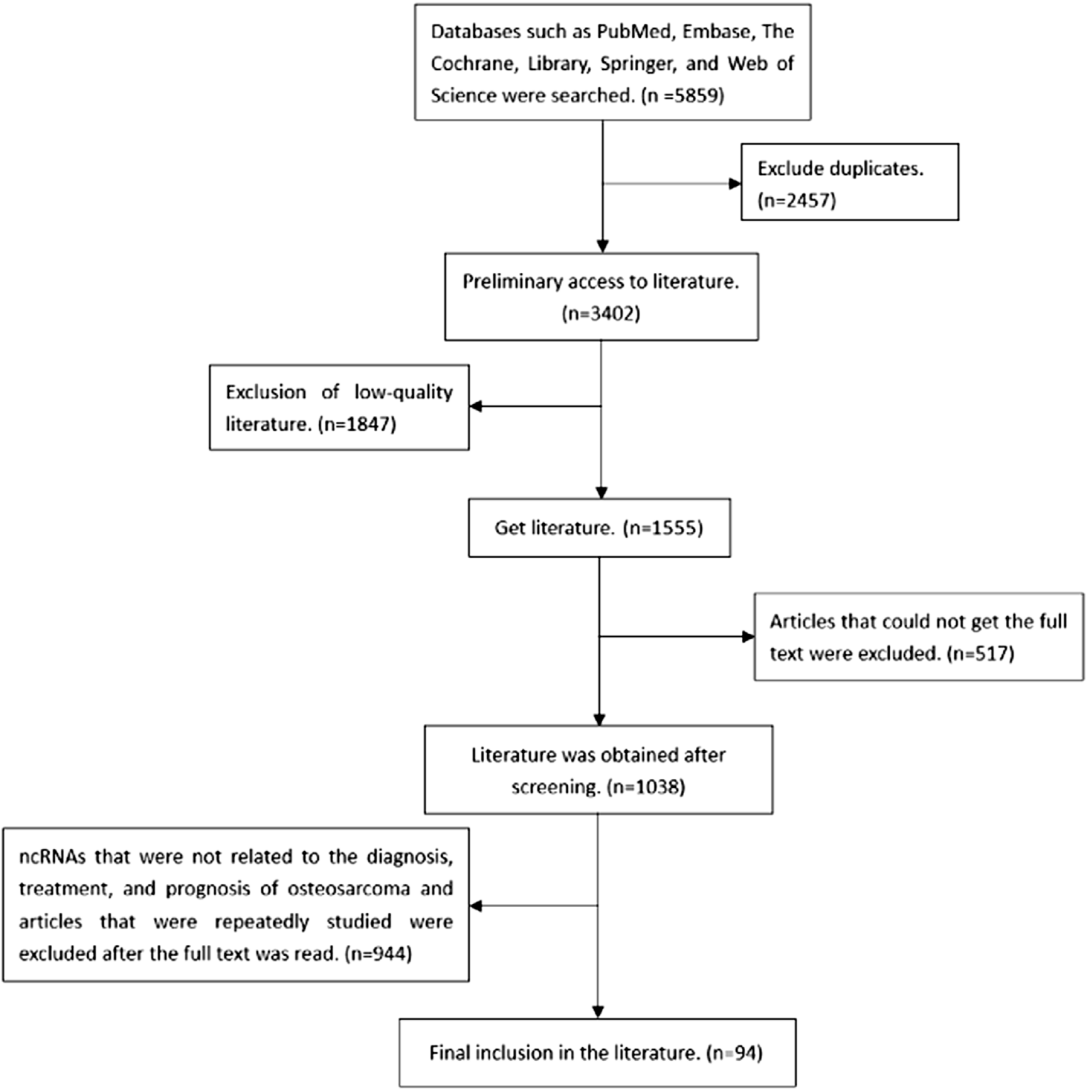
Flow chart of literature selection and collection.

At present, the researches focused on ncRNAs tumor markers and relevant action pathways have revealed the important roles in the occurrence, metastasis and prognosis. These biomarkers can help to determine the accuracy of the diagnosis, decide on the appropriate treatment time, make personalized treatment plans, and explore new therapeutic targets. Many ncRNAs biomarkers and corresponding protein products have been reported to be overexpressed or under-expressed in OS, such as miR-223 (Xu et al., 2013), COP9 signalosome subunit 3 (COPS3) (Zhang et al., 2018), lncRNA small nucleolar RNA host gene 4 (lncRNA SNHG4) (Xu R et al., 2018), miR-142 (Shabani et al., 2019), etc. Although the reports on types and functions of ncRNAs in OS are still limited, they might express some diagnostic, stage, treatment and prognosis targets. ncRNAs such as miRNAs, lncRNAs, circRNAs studied before were summarized in this review. miRNA is a small ncRNA molecule, which mainly regulates gene expression by degrading mRNA or inhibiting the translation process after transcription. The discovery of lncRNA mainly comes from microarray technology, the second generation high-throughput transcriptome sequencing technology, single cell sequencing technology, etc., and plays an important role in maintaining the activities of living cells. circRNA is a wide range of ncRNA that is stable in nature, highly conservative and specific. In this paper, by searching the relevant databases, we searched the ncRNA related to the occurrence and development of OS, and expounded its role in the occurrence and development of OS. The specific literature screening process is shown in Figure 1. The specific roles of some of the relevant ncRNAs are presented in Table 1.

**TABLE 1:** Identification of NcRNA Tumor Biomarkers analyzed in multiple studies.

Tumor biomarker	Deregulation	Genes/proteins affected	OS cases	Controls	Observation in OS	Ref
circ_0008717	Overexpression	miR-203/Bmi-1	45	45	Correlation with the patient’s poor prognosis and lung metastasis	(Zhou et al., 2018; Lei and Xiang, 2020)
circ_CDR1as	Overexpression	CDR1as/miR-7	38	18	Associated with tumor size, Enneking staging, and distant metastasis	Xu R et al. (2018)
circ_TADA2A	Overexpression	miR-203a-3p	10	10	Related to migration, invasion and proliferation	Wu et al. (2019)
circ_103801	Overexpression	miR-338-3p/HIF-1/Rap1/PI3K-Akt	—	—	Related to the proliferation, migration and invasion of tumor	Li S et al. (2021)
circ_0001721	Overexpression	miR-758/TCF4/Wnt/β-catenin	51	51	Related to cell resistance and growth	Guan et al. (2021)
circ_0000502	Overexpression	miR-1238	63	63	Relate to tumor proliferation, migration, invasion and apoptosis	Qi et al. (2018)
circ_MTO1	Low-expression	miR-630	44	44	Associate with Enneking stage and/or pathological fracture, as well as neoadjuvant treatment	Shi et al. (2021)
circ_0008932	Overexpression	miR-145-5p	50	50	Closely related to the proliferation, migration, invasion and apoptosis of OS cells	Cao and Shu, (2021)
circ_PVT1	Overexpression	Gene ABCB1	80	20	Increased risk for chemotherapy resistance	Kun-Peng et al. (2018)
circ_LRP6	Overexpression	KLF2/APC	50	50	Associated with proliferation, migration, invasion	Zheng et al. (2019)
circ_001621	Overexpression	miR-578/CDK4/MMP9	30	—	Related to proliferation and migration	Ji et al. (2020)
circ_0001721	Overexpression	miR-569 and miR-599	52	52	Closely relate to that poor prognosis and clinical severity of the patient	Li L et al. (2019)
circ_hsa_circ_0003074	Overexpression	—	60	60	Closely related to clinical characteristics, such as tumor size, lung metastasis, enneking stage, and chemotherapy resistance	Lei and Xiang, (2020)
circ_0081001	Overexpression	miR-494-3p/TGM2	35	28	Can affect tumor metastasis and chemotherapy sensitivity	Wei et al. (2021)
circ_0007534	Overexpression	Bcl-2/caspase-3	57	57	Relate to that proliferation, migration, invasion and apoptosis of tumor cells	Li and Li, (2018)
circ_001569	Overexpression	miR-185-5p/FLOT2	20	20	Related to proliferation, migration, invasion, EMT, tumor size, Enneking stage (or TNM stage), and lung metastasis	Xiao et al. (2020)
has_circ_0009910	Overexpression	miR-449a/IL6R	30	30	Promote OS cell growth and inhibiting apoptosis	Deng et al. (2018)
circ_UBAP2	Overexpression	miR-2043p/HMGA2	42	42	Associated with OS cell proliferation, invasion and migration, and negatively correlated with overall survival	Ma et al. (2021)
circ_HIPK3	Overexpression	miR-637/HDAC4	12	12	Has a positive correlation with that total survival time of the patient	Wen et al. (2021)
circ_0021347	Low-expression	B7-H3	35	35	Negatively correlated with TNM staging, positively correlated with patient survival, and negatively correlated with B7-H3	Wang et al. (2019)
hsa_circ_0000658	Low-expression	miR-1227/IRF2	60	60	Differentiation grade and distant metastasis	Jiang and Chen, (2021)
miR-21-5p	Overexpression	Wnt/β-catenin/PTEN/Akt	1	1	Positively correlated with the differentiation grade and distant metastasis of the tumor	Wu H et al. (2021)
miR-124	Low-expression	—	114	50	Lower 5-year survival and disease-free survival	Cong et al. (2018)
miR-21	Overexpression	—	65	30	Might be a good candidate for a therapeutic target, and a potential biomarker for the prediction of chemotherapeutic sensitivity and prognosis	Yuan et al. (2012)
miR-21	Overexpression	—	94	94	Correlated with the pathological stage, tumor grade, and lung metastasis	Zhao H et al. (2019)
miR-21	Overexpression	—	69	69	Closely related to the therapeutic effects of OS, and can be used as a potential biomarkers and therapeutic targets for the diagnosis and prediction of OS	Hua et al. (2017)
miR-143	Overexpression	ERK/MAPK	2	—	Relate to tumor invasiveness	Wang et al. (2014)
miR-100	Low-expression	PI3K/AKT/MAPK/ERK/IGFIR	20	20	Associated with tumor proliferation, migration, invasion and chemotherapy resistance	Liu et al. (2016)
miR-140	Low-expression	HDAC4	10	—	Overexpression of miR-140 inhibits the proliferation and invasion of OS cells, and promotes their apoptosis	Song et al. (2009)
miR-217	Low-expression	SIRT1	42	37	Relate to OS cell proliferation, migration and invasion	He et al. (2019)
miR-646	Low-expression	FGF2	64	64	Relate to OS cell proliferation, migration and invasion	Sun et al. (2015)
miR-223	Low-expression	—	112	50	Related to the metastasis of OS and could be used as a potential diagnostic and prognostic biomarker	Dong et al. (2016)
miR-382-5p	Low-expression	VEZF1	20	20	Affect OS cell proliferation, migration, aggregation, invasion, apoptosis	Wu Z et al. (2021)
miR-191	Overexpression	checkpoint kinase 2	—	—	Affect OS cell proliferation	Huang et al. (2015)
miR-143-3p	Low-expression	FOSL2/MAPK7	20	20	Relate to OS cell proliferation, migration and invasion	Sun et al. (2018)
miR-145	Low-expression	MMP16/VEGF	31	35	Affect that invasion and migration of OS cells	Chen et al. (2015)
miR-145-5p	Low-expression	E2F transcription factor 3	20	10	Affect the proliferation and progression of tumor cells	Li H et al. (2020)
miR-335	Low-expression	SNIP1	37	37	Related to the migration and invasion of tumor cells	Xie et al. (2019)
miR-221	Overexpression	PTEN/PI3K/AKT	108	108	Associated with overall survival and its high expression predicts a poor prognosis	Yang et al. (2015)
miR-106a	Overexpression	VNN2	18	18	Can regulate the proliferation and invasion of OS cells	Chen et al. (2018)
miR-210	Overexpression	FGFRL1	54	54	Associated with larger tumor volumes, poor preoperative chemotherapy response, metastases	Liu et al. (2018)
miR-27a	Overexpression	SIRT1/Wnt/β-catenin/MAP2K4	166	60	Associated with invasion, proliferation, metastasis	Tang et al. (2015)
miR-646	Low-expression	FGF2	64	64	Low expression is associated with metastasis, and its overexpression inhibits cell proliferation, migration, and invasion	Sun et al. (2015)
miR-382-5p	Low-expression	VEZF1	20	20	Related to the proliferation, invasion, migration and apoptosis of OS cells	Wu H et al. (2021)
miR-191	Overexpression	checkpoint kinase 2	—	—	Forced expression of miR-191 can promote the proliferation of OS cells, while miR-191 antisense oligonucleotides block cell proliferation	Huang et al. (2015)
miR-410	Low-expression	VEGF	—	—	The overexpression of miR-410 exerted greater inhibition on the expression of VEGF	Chen et al. (2017)
miR-19a	Overexpression	JAK2/STAT3	—	—	Closely related to cell proliferation and apoptosis	Chen and Chen, (2020)
lncR-FTX	Overexpression	miR-320a/TXNRD1	25	25	Knocking out FTX can inhibit OS cell proliferation and migration, and promote apoptosis	Huang et al. (2020)
lncR-91H	Overexpression	CDK4	—	—	Knock-out of 91H inhibits the occurrence of OS by inducing methylation of CDK4 promoter *in vitro* and *in vivo*	Cheng et al. (2021)
lncR-BCAR4	Overexpression	—	168	168	Significantly correlated with the overall survival rate, clinical stage and distant metastasis	Ju et al. (2016)
lncR-FGFR3-AS1	Overexpression	—	62	62	Related to tumor volume, Enneking staging, metastasis, and survival	Sun et al. (2016)
lncR-GNAS-AS1	Overexpression	miR-490-3p	112	1	Patients with high lncR- GNAS-AS1 expression represented shorter overall survival and was an independent prognostic predictor of OS	Mi et al. (2021)
lncR-HIF2PUT	Overexpression	HIF2	30	30	Relate to proliferation, migration and invasion of OS patients	Zhao H et al. (2019)
lncR-HotTIP	Overexpression	PTBP1/KHSRP	20	20	Relate to that proliferation, invasion and migration of OS cells	Yao et al. (2021)
lncR-HULC	Overexpression	miR-372-3p/HMGB1	32	32	Overexpression of HULC or knockdown of miR-372-3p promotes the proliferation, migration, and invasion of OS cells and induces apoptosis	Li L et al. (2020)
Malat-1	Overexpression	—	—	—	Relate to chemotherapy resistance of OS cells	Liu et al. (2021)
lncR-UCA1	Overexpression	miR-513b-5p/E2F5	—	—	Related to the proliferation, migration and invasion of OS cells	Zhang et al. (2021)
lncR-DLX6-AS1	Overexpression	miR-641/HOXA9	40	40	Relate to OS cell proliferation and metastasis	Zhang N et al. (2019)
lncR-TUSC7	Low-expression	miR-181a/RASSF6/miR-211	45	45	Overexpression inhibits the proliferation, migration and invasion of OS cells, and promotes the apoptosis of cells *in vitro* and *in vivo*	Zhao et al. (2021)
lncR-MEG3	Low-expression	miR-361-5p/FoxM1	78	126	Associated with the synergistic regulation of miR-361-5p/FoxM1 on the proliferation, migration and apoptosis of OS	Li Y et al. (2020)
lncR-SNHG3	Overexpression	miR-196a-5p	127	127	Related to overall survival rate of patients and tumor size	Chen et al. (2019)
lncR-SNHG4	Overexpression	miR-224-3p	136	40	Promote tumor growth and represent a poor prognosis	Xu B et al. (2018)
lncR-CCAT2	Overexpression	GSK3β/β-catenin	50	50	Positive correlation with tumor size, stage and overall survival rate	Ruan and Zhao, (2018)
lncR-SOX21-AS1	Overexpression	miR-7-5p/IRS2	—	—	High expression leads to the proliferation, migration and invasion of OS cells	Chen and Chen, (2021)
lncR-DLX6-AS1	Overexpression	miR-641/HOXA9	40	40	Related to TNM stage, clinical stage and distant metastasis of OS	Zhang H et al. (2019)
lncR-Sox2OT-V7	Overexpression	miR-142/miR-22	—	—	Associated with chemotherapy resistance	Zhu et al. (2020)
lncR-NR-036444	Low-expression	—	60	—	May be used as a biomarker to distinguish the chemotherapy sensitivity and judge the prognosis of OS.	Zhu and Zhang, (2017)
lncR-SNHG4	Overexpression	miR-224-3p/DOCK7	136	40	Positively correlated with tumor volume and negatively correlated with overall survival rate	Xu B et al. (2018)
lncR-XIST	Overexpression	miR-153-SNAI1	30	30	Relate to migration, invasion and EMT of OS cells	Wen et al. (2020)
lncR-DANCR	Overexpression	miR-149/MSI2	109	109	Relate to TNM stage	Zhang et al. (2020)
lncR-FOXD2-AS1	Overexpression	—	20	20	Negatively correlated with overall survival. Positive correlation with migration and invasion	Zhang N et al. (2019)
lncR-TTN-AS1	Overexpression	miR-134-5p/MBTD1	—	—	Positively correlated with chemotherapy resistance and tumor volume	Fu et al. (2019)
lncR-BC050642	Overexpression	c-myc	97	97	Related to the clinical stage and cell viability of the patient	Yang et al. (2019)
lncR-01614	Overexpression	miR-520a-3p/SNX3	10	0	Positive correlation with migration, invasion and tumor size of OS cells	Cai et al. (2021)
lncR-SNHG16	Overexpression	miR-488/ITGA6	10	10	Promoted migration, invasion and EMT of OS by sponging miR-488 to release ITGA6	Bu et al. (2021)
lncR-NNT-AS1	Overexpression	—	126	126	Related to the migration and invasion of tumor cells	Ye et al. (2018)
lncR-BCAR4	Overexpression	GLI2-dependent gene	60	60	Positive correlation with tumor size, migration, invasion and invasion stage. Negatively correlated with overall survival	Chen et al. (2016)
lncR-MALAT1	Overexpression	miR-150-5p/VEGFA	—	—	Induction of angiogenesis	Vimalraj et al. (2021)
piR-39980	Overexpression	SERPINB1/MMP-2	2	—	Positively correlated with the proliferation, migration and invasion of OS cells	Das et al. (2020)
hsa_piR-006613	Low-expression	FN1 mRNA	2	—	Positively correlated with proliferation and migration	Cui et al. (2022)
lncR-NR-136400	Low-expression	TUSC5	4	3	Promoting the proliferation and migration of OS cells	Liu et al. (2020)
ceR-IGF-1	Overexpression	miR-29s/VEGF	1	1	Promote angiogenesis	Gao et al. (2016)
circ_0001785	Overexpression	miR-1200/HOXB2/PI3K/Akt	—	—	Promote proliferation	Li L et al. (2019)
circ_DOCK1	Overexpression	miR-339-3p/IGF1R	70	70	Increased oncogenicity *in vivo* and malignant transformation *in vitro*	Li S et al. (2021)
circ_EPSTI1	Overexpression	miR-892b	50	50	Promote migration and invasion of OS cells	Tan et al. (2020)
circ_0102049	Overexpression	miR-1304-5p/MDM2	76	76	Positively correlated with the tumor volume and lung metastasis of the patient, and negatively correlated with the overall survival rate	Jin et al. (2019)
lncR-HCG9	Overexpression	miR-34b-3p/RAD51	15	15	Positively correlated with proliferation, migration and invasion	Wang et al. (2021)
lncRNA-SNHG16	Overexpression	miR-1285-3p	50	50	Positively correlated with proliferation, migration, invasion, apoptosis	Xiao et al. (2021)
PIK3CA	Overexpression	—	59	63	High expression means that patients have a higher risk of OS	He et al. (2013)
iNOS	Overexpression	Wnt/β-Catenin	45	45	INOS is closely related to the formation of OS, and inhibition of iNOS will affect the effect of β-protein	Chu et al. (2021)
IDH1	Low-expression	—	44	16	Upregulation can significantly reduce the invasion and migration activity of OS	Hu et al. (2014)
TP53	mutated	—	425	—	Oncogenic function of mutant TP53 maintains tumor cell proliferation and growth in OS	Tang et al. (2019)
RECQL4	mutated	—	18	12	Necessary for normal OS amplification and OS formation	Maire et al. (2009)
DLG2	mutated	—	31	—	Associated with susceptibility to OS	Shao et al. (2019)

In this review, the roles of ncRNAs who had the potential as biomarkers in the diagnosis, stage, treatment, and prognosis of OS were discussed in depth, which has great potential to provide reference for relevant studies.

## Diagnostic biomarkers

Diagnosing OS at the early stage can cure diseases faster and earlier, significantly improve the prognosis of patients. Abnormal expression of ncRNA biomarkers can be used as potential biomarkers for diagnosis of OS. For miRNAs, there were significant differences in serum and plasma levels of miR-21 between patients with OS and healthy people, which found can be used as biomarkers of OS. Studies have shown that miR-21 was over-expressed in OS tissues and cell lines, and it had great potential as a biomarker for OS diagnosis. Cong et al. (2018) found that the serum miR-124 level in patients with OS was significantly lower than that in the periostitis group and the healthy control group. The level of miR-124 normalized after tumor resection, and the area under the area under curve (AUC) for serum miR-124 was 0.846, with sensitivity of 79.8% and specificity of 86.0%, which proved that miR-124 had the potential to be the biomarker for diagnosis of OS. Wang et al. (2014) found that compared with the corresponding para-carcinoma tissue, the miR-143 level was significantly decreased in OS, which proved that miR-143 can be used as diagnostic biomarker of OS.

Fraxetin (FXT), had been reported to be associated with the development of various tumors (Yao et al., 2022). Through examining the expression of FTX in 25 OS and the adjacent tissues, Huang et al. (2020) found that FTX were significantly upregulated in OS tissues, and knocking out FTX gene could inhibit the survival rate, invasion, and migration force of OS cells, as well as promote the apoptosis of OS cells. LncRNA can not only directly regulate OS cells, but also affect the growth, proliferation, apoptosis and invasion of OS cells through the regulation of mRNA or miRNA, playing a key regulatory role in the occurrence and development of OS. The research studied by Li et al. (2017) found that lncRNA can be regulated in the occurrence and development of OS by competing endogenous RNAs (ceRNA), Wnt/β-Catenin and other pathways. In short, lncRNA can be served as biomarkers for early screening of OS, but their specificity and sensitivity need to be further verified clinically.

**FIGURE 2:**
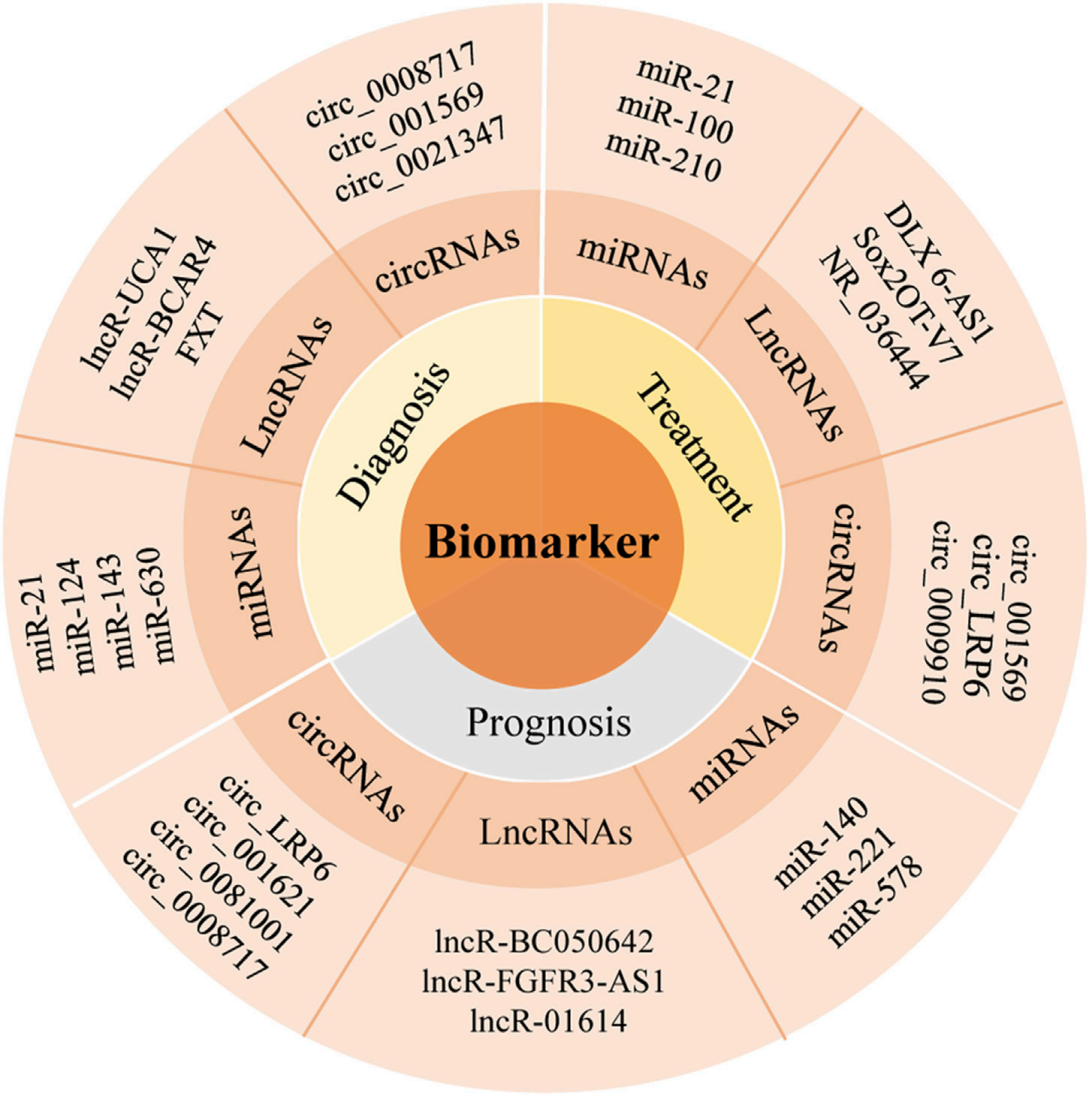
The role of partial ncRNAs in OS.

When it comes to circRNAs, recently more and more studies have shown that circRNAs can be used as a kind of biomarkers in the early diagnosis of OS, with high accuracy and specificity. Zhou et al. (2018) reported that circ_0008717 owned high expression in OS tissues, compared with the corresponding paracancerous tissues (Figure 2). Molecular sponge could bind to miR-203 to exert its carcinogenic effect. Its AUC was 0.782 (95% CI: 0.682–0.862), sensitivity was 0.80, and specificity was 0.73, which could be used as a potential biomarker for the diagnosis of OS. The commonly used biomarkers for clinical OS diagnosis are serum alkaline phosphatase (AKP) and lactate dehydrogenase (LDH), but the comparison of diagnostic efficacy between these biomarkers and OS biomarkers has not been reported. Lei and Xiang (2020) found that hsa_circ_0003074 was highly expressed in plasma of OS patients and had a high efficacy in distinguishing OS patients from healthy volunteers, with the AUC of 0.93, higher than LDH (AUC = 0.83) and alkaline phosphatase (ALP) (AUC = 0.88). Thus, circRNAs also had the potential to be a diagnostic biomarker, which need further studies.

It can be observed that many articles have proved that ncRNAs also has the potential as a diagnostic marker. In addition to the function in diagnosis, the levels of OS ncRNAs biomarkers also play an important role in determining the disease stage of OS at diagnosis. Abnormal expression of OS related genes and biomarkers can induce the activity, proliferation and differentiation of osteoblasts. The abnormal degree can directly reflect the pathological staging of patients and affect the prognosis. Enneking staging has become an important basis of OS surgical staging which can better remind the prognosis, and help to select an appropriate treatment. However, research on the relationship between ncRNAs biomarkers and tumor staging is still very lacking. A variety of ncRNAs biomarkers are closely related to the clinical stage of Enneking and have been widely used to determine the clinical stage of OS. The more malignant the lesion is, the higher Enneking stage and higher possibility of distant metastasis the tumor have.

It was found that the expression of lncRNA GNAS antisense (lncRNA GNAS-AS1) could significantly increase in OS cells and tissues, which were positively correlated with Enneking stage and distant metastasis. Its high expression often predicted the short overall survival time (Mi et al., 2021). Through regulating miR-490-3p, lncRNA GNAS-AS1 could play a major role in cell proliferation, migration and invasion, which could be an independent prognostic predictor of OS, providing a new therapeutic strategy for OS (Mi et al., 2021). The expression of circRNA-mitochondrial tRNA translation optimization 1 (circ_MTO1) in OS is low, its expression level is significantly correlated with Enneking staging, and its high expression in the tumor meant that the Enneking staging was lower in OS patients who had better neoadjuvant chemotherapy response and longer disease-free survival (DFS) (Shi et al., 2021). Except for the above, the increased expression of circ_001569 in OS was positively related to tumor size, Enneking stage or Tumor Node Metastasis (TNM) stage and lung metastasis (Xiao et al., 2020). The results reported by Wang et al. (2019) showed that circ_0021347 was significantly downregulated in OS tissues and cell lines, negatively correlated with TNM staging and positively correlated with patient survival. circ_0021347 can target B7 Homolog3 (B7-H3), which showed a strong negative correlation with the expression of B7-H3 in OS and exerted anti-cancer effect by negatively regulating the expression of B7-H3.

In a word, the emergence of ncRNAs biomarkers in patients provides a reliable non-invasive method for the diagnosis and clinical staging of OS. However, when exploring new serum biomarkers in the future, the sensitivity and specificity of existing biomarkers should not be ignored, so as to provide accurate guidance information for clinical practice.

## Therapeutic biomarkers

Neoadjuvant chemotherapy is the standard treatment at present, and its survival rate has been greatly improved. However, the survival rate of patients with lung metastasis and chemotherapy resistance is still very low. Although the introduction of neoadjuvant chemotherapy has greatly improved the 5-year survival rate of OS, a large number of patients still have poor response to chemotherapy. Even after surgical resection and chemotherapy, there still exist a high risk of local recurrence or distant metastasis, leading to poor prognosis. It is necessary to develop new therapeutic methods for OS clinically, which require us to clarify the molecular mechanisms of the pathogenesis and development of OS. The screening of new molecular markers is important for the prognosis and treatment of OS.

The ncRNAs have good stability, which can be used as potential cancer biomarkers and treatment targets (Rong et al., 2017). Previous studies have explained the mechanism of OS from multiple drug resistance-related genes, miRNAs, circRNAs and other aspects. Significant changes of miRNAs expression profile in drug-resistant OS cells indicate that miRNAs can participate in the development of drug resistance by regulating various targets and signaling pathways. The treatment based on miRNAs mainly included blocking the expression of oncogenic miRNAs and restoring the expression of tumor-inhibiting miRNAs genes. For instance, miR-21, which was highly expressed in many cancer types, had higher expression level in the sera of OS than that in the healthy, which has been used clinically as a biomarker of chemotherapy sensitivity and prognosis (Yuan et al., 2012; Hua et al., 2017; Zhao H et al., 2019). Related studies have proved that transforming growth factor-β1 (TGF-β1) inhibitor treatment reduced the inhibitory effects of miR-21 knockdown on OS cell proliferation. miR-21 inhibition may inhibit OS cell proliferation by targeting PTEN and regulating the TGF-β1 signaling pathway (Hu et al., 2018). In addition, the miRNAs profile is associated with tumor response which can be a preferred tool for predicting tumor susceptibility to treatment.

With the deepening understanding of genetic biomarkers, the therapeutic effect of lncRNAs has become a hot research topic in recent years, which is superior to known protein encoded gene biomarkers in predicting drug responses. At present, the abnormal expression of lncRNAs has been found in many patients. These abnormal expressions can affect the processes of drug outflow, apoptosis, DNA repair, cell cycle, proliferation, autophagy, etc. At the same time, these abnormal expressions participate in the chemotherapy resistance of OS by regulating the expression of different target genes and related signaling pathways. Distal-less homeobox 6 antisense 1 (DLX 6-AS1), which was highly expressed in OS patients, exerted its capability of inhibiting the proliferation, invasion and metastasis of OS cells by targeting the miR-641/homeobox protein Hox-A9 (HOXA9) signaling pathway (Zhang H et al., 2019). Upregulation of SOX2 overlapping transcript lncRNA transcript variant 7 (Sox2OT-V7) in OS can directly target miR-142/miR-22 to inhibit its expression, especially in OS tissues and cell lines that were resistant to chemotherapy. When knocking out Sox2OT-V7 in OS cells, the drug-resistant U2OS/Dox cells can be re-sensitive to chemotherapy drugs (Zhu et al., 2020). Small nucleolar RNA host genes 4 (SNHG 4) can play a role in the occurrence and development of OS through the miR-224-3p/DOCK7 pathway, which provide a new method in the treatment of OS (Xu B et al., 2018). Lee A. M. et al. (2021) identified a positive correlation between the expression of lncRNA and anti-sense non-coding RNA in the INK4 locus (ANRIL) and the resistance of two therapeutic drugs for OS, cisplatin and doxorubicin. It was found that the drug resistance of MG-63/DXR cells transfected with lncRNA NR_036444 decreased significantly, the proportion of cells in the G (1) phase increased. The proportion of cells in the later stage of apoptosis also increased, which might play an important role in regulating the drug resistance to doxorubicin, and might become a useful biomarker to evaluate chemotherapy sensitivity and predict prognosis of OS in the future (Zhu and Zhang, 2017).

CircRNAs can play positive or negative regulatory roles in the occurrence and development of OS. In the fact, circRNAs can further participate in the regulation of chemotherapy resistance and metastasis of OS through “sponging” miRNAs as a tumor activating or inhibiting factor. The treatment based on miRNAs mainly included blocking the expression of oncogenic miRNAs and restoring the expression of tumor-inhibiting miRNAs genes. The carcinogenic effects of circ_0009910 were partially dependent on the JAK2/STAT3 pathway, which was involved in the apoptosis and proliferation of cells, thus affecting the progression of OS (Richardson et al., 2010). And circ_001569 may promote resistance through the Wnt/β-catenin pathway (Wu et al., 2014).

Drug resistance is the main limiting factor for the effectiveness of cancer treatment. Some drugs can quickly relieve the tumor, but on the one hand, they can also produce resistance, thus increasing the difficulty of treatment (Vasan et al., 2019). At present, more and more ncRNAs have been found to be associated with drug resistance of OS (Raei et al., 2021). The reversal of targeted ncRNAs is likely to be a potential method for reversing drug resistance of OS, which requires us to consider how to select the key ncRNAs from a large number of candidates ncRNAs. In the future, we should actively carry out clinical trials or transformation research based on targeted ncRNAs therapy, clarify the detailed mechanism of ncRNAs in OS drug resistance, and further apply them to clinical treatment of OS.

## Prognostic biomarkers

OS has a poor prognosis due to chemo-resistance and/or metastases. ncRNAs biomarkers of OS can affect the prognosis in many ways. Clarifying the relationship between ncRNAs biomarkers and the prognosis of OS, can help to optimize the current treatment plan and choose the right time of surgery and chemotherapy.

A variety of miRNAs also have great significance for the prognosis and progression of OS. The expression of miR-140 was related to the chemotherapy sensitivity of OS xenografts, which was involved in a wide range of chemotherapy resistance mechanisms by inhibiting HDAC4-mediated decrease in cell proliferation in G (1) and G (2) stages and participating in chemotherapy resistance. miR-140 could inhibit the proliferation of U2OS cells, but its inhibitory effect on MG-63 cell was weak (Song et al., 2009). miR-223 has proved to be a potential prognostic marker for a variety of cancers in the past. Recently, some scholars have observed its function in OS. They found that miR-223 may be related to OS metastasis, and it has great potential as a potential biomarker for diagnosis and prognosis of OS (AUC:0.926) (Dong et al., 2016).

LncRNAs can play important roles in drug sensitivity and cancer metastasis. lncR-BC050642 was significantly increased in OS tissues and cell lines, playing an important role in promoting the proliferation of OS cells, who can also be an independent biomarker of OS prognosis (Yang et al., 2019). In OS tissues, the significantly upregulated lncR-01614 indicated a worse prognosis for patients. After knocking out lncR-01614, the proliferation, invasion and metastasis of OS cells could be inhibited. This effect was achieved through the miR-520a-3p/SNX3 regulatory axis, which had the potential to be a new clinical prognostic marker for OS (Cai et al., 2021). Titin-antisense RNA1 (TTN-AS1) could act as a carcinogen, playing a role in cancer through the miR-134-5p/MBTD1. Its high expression was also significantly related to the poor prognosis, and it had a potential to be a biomarker (Fu et al., 2019).

**FIGURE 3:**
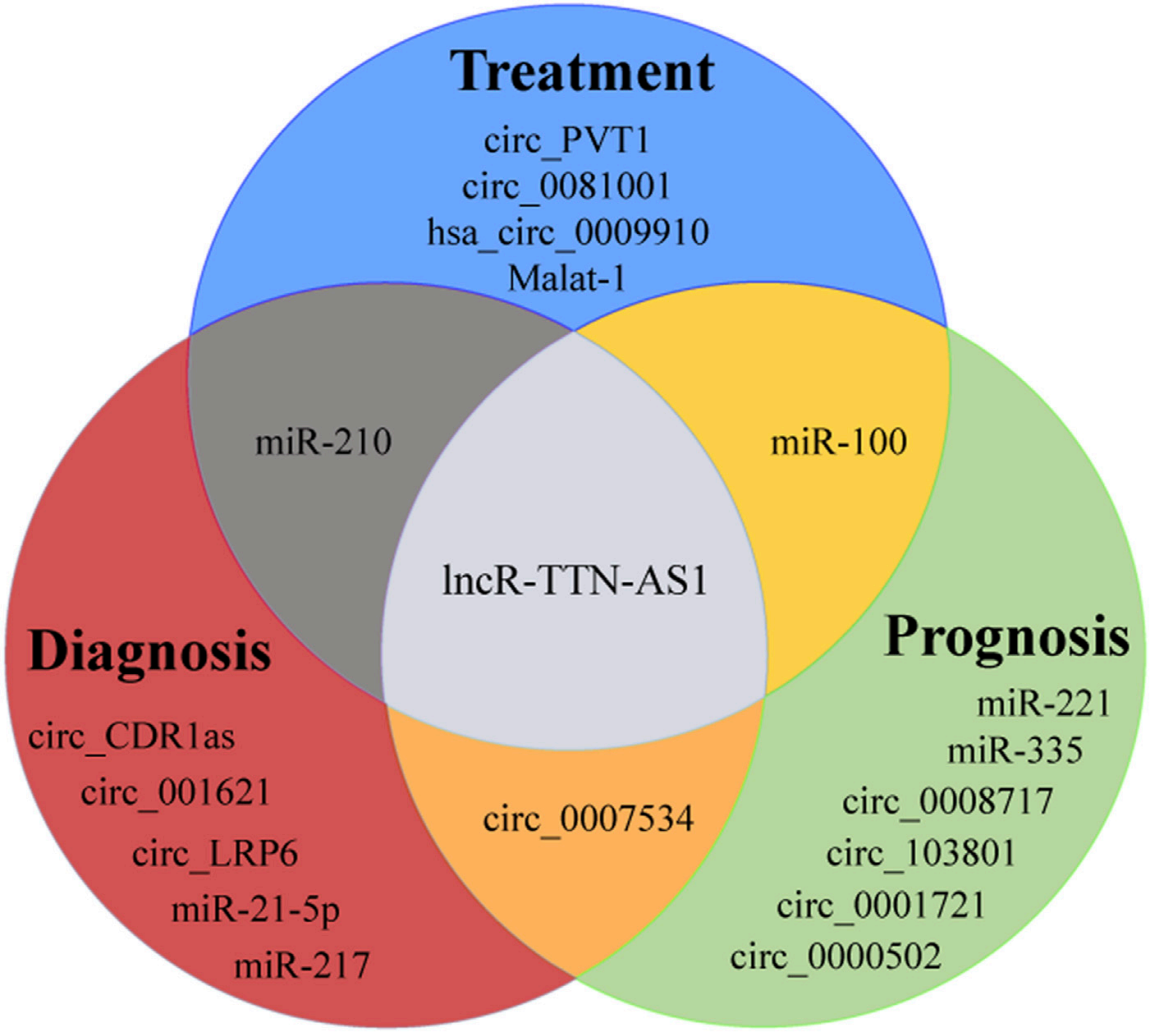
Crosstab of the roles of selected ncRNAs in osteosarcoma.

CircRNA LRP6 (circ_LRP6) was highly expressed in OS, which can promote the progress of OS by inhibiting the expression of APC and KLF2. Its high expression usually indicated the short-term disease-free survival time and overall survival time (Zheng et al., 2019) (Figure 2). Studies have established an experimental system to study the *in vivo* and *in vitro* effects of the interaction between circ_001621/miR-578/VEGF, and the results show that circ_001621 played an important role in OS, and can increase the malignant degree, directly inhibit the expression of miR-578, significantly upregulate the expressions of VEGF, cyclin dependent kinase 4 (CDK4) and matrix metalloprotein 9 (MMP-9) in MG-63 and U2OS cells. Further it can promote the proliferation and migration of OS cells and provide a new therapeutic target for advanced OS (Ji et al., 2020) (Figure 3). It was found that circ_0008717 and lung metastasis are independent prognostic factors through Cox multivariate regression analysis. After knocking down the expression of circ_0008717, the proliferation, migration and invasion of tumor cells can be reduced, and apoptosis can be promoted (Shen et al., 2022). circ_0081001 was another potential biomarker of OS prognosis, which was selected from chemotherapy and chemotherapy-sensitive OS cell lines, showing high levels in advanced OS, chemotherapy-resistant and lung metastasis (Wei et al., 2021) (Figures 2, 3).

Although some ncRNAs as prognostic biomarkers have been identified in OS, the outcome was not fully validated. As there have been some studies on the prognostic markers of OS, there is no practical application. The function of biomarkers for the diagnosis and prognosis of OS, especially patients with metastasis, may be an urgently needed tool for early diagnosis and identifying potential therapeutic targets.

## Perspectives and future opportunities

The early symptoms of OS are not typical. It is easy to metastasize, and the treatment often lagging behind with poor effect. Although the diagnosis and treatment of OS have made continuous progress in recent years, the 10-year survival rate of patients with metastatic OS is still less than 20% (Yang et al., 2020). As a result, new early diagnosis and treatment methods of OS are urgently needed. Biomarkers refer to biochemical indicators that can mark changes or possible changes in the structure or function of systems, organs, tissues, cells, and subcellular. The development of biomarkers provides new insights for the early diagnosis and treatment of diseases, which can assist clinicians in initial diagnosis especially for patients with metastatic OS, guide the treatment method, judge disease stages, evaluate the safety and effectiveness of new drugs or therapies in target population, providing a series of key genes and approaches for elucidating the molecular mechanism of OS. Therefore, it is urgent to search for useful biomarkers and therapeutic targets for clinical application. ncRNAs can be produced in the early stage of disease, which is an important part of epigenetics research. It can be used as a key regulatory factor to regulate the expression of related genes and participate in the processes of cell development, differentiation, proliferation, transcription, post-transcriptional modification, apoptosis, and cell metabolism (de la Fuente et al., 2012). Moreover, it can naturally connect related genetic networks to affect various basic protein effect factors that drive specific cellular biological responses and determine cell fate (Wei et al., 2017). As a carcinogen or anti-cancer factor, it plays an important role in the occurrence and development of a variety of cancers, with a certain stability and richness. ncRNAs can enter the circulatory system, which is expected to become a potential biomarker of OS (Slack and Chinnaiyan, 2019).

The application of these ncRNAs in the research and development of anti-OS drugs, related therapeutic targets and biomarkers in the field of OS was discussed in detail in this paper. At present, the newly discovered tumor biomarkers are one of the hot spots in oncology research. The research on ncRNAs-encoded peptides or proteins has opened up a new research field for the diagnosis and treatment of tumors. As for OS, ncRNAs can be produced in the early stage of the disease, with certain stability and richness. When them entered the circulatory system, they can play an important role in cell function and affect its clinical manifestations to a certain extent. It is expected to become an effective diagnosis and treatment method. However, current research on ncRNAs also has the following problems. Firstly, the related research on ncRNAs and OS is limited and not in-depth. The current research mainly focuses on basic *in vitro* experiments. Further animal experiments and clinical trials are needed to confirm it in the future. Secondly, the targeted anticancer drugs of ncRNAs can weaken or even eliminate drug resistance of the patients, and further enhance the therapeutic effect. However, these drugs still have the disadvantages of insufficient specificity and utilization rate. The absorption and biodistribution of some drugs still need further solution. There is still a long way to go before the approval and commercialization of ncRNAs-targeted anticancer drugs. Thirdly, ncRNAs have many targets and complex regulatory networks. At present, most research on ncRNAs are independent and scattered. The lack of specificity in the diagnosis and treatment of OS targeting ncRNAs still needs to be resolved. Finally, there still exist problems such as small sample size in the current research on the relationship between ncRNAs and OS, and lacking of epidemiological evaluation and functional exploration of candidate biomarkers. Only a few ncRNAs have been fully studied, and the possibility of its clinical application is still uncertain. In addition, most ncRNAs cannot be detected by standard methods such as quantitative PCR. It is an urgent to find new detection optimization methods and advanced laboratory platform conditions.

In summary, the expression of the biomarker is significantly related to the tumor size, distant metastasis, TNM stage, and Enneking surgical stage of the patients, which has the ability to distinguish OS from the healthy. It is important to look for a reliable clinical biomarker in the field of the early diagnosis and treatment of OS. These biomarkers are helpful to clarify the molecular mechanisms related to OS, and may become new targets for OS diagnosis and treatment. The higher the level of biological evolution, the higher the proportion of ncRNAs in the genome. Constituting almost 60% of the transcriptional output in human cells, ncRNAs have been shown to regulate cellular processes and pathways in developmental and pathological contexts (Anastasiadou et al., 2018). Therefore, searching for more types of ncRNAs and its more extensive and diverse biological functions and action mechanisms are our current main tasks. The role of these ncRNAs in the proliferation, occurrence and development of OS cells should be deeply studied, so as to better predict and diagnose OS, provide meaningful theoretical guidance for individualized treatment and drug research. In the future, ncRNAs may continue to better help researchers and clinicians find molecular features, help in differential diagnosis, personalized treatment and determine prognosis et al. Although the applications of ncRNAs are still at an early stage, it deserves expectation can be put into clinical practice especially those candidates that can be readily detected. In the follow-up study, we would further verify the efficacy of diagnosis, treatment and prognosis of OS in large samples, and combine multiple ncRNAs to build a model to assist the diagnosis of clinical OS. Benign biomarkers should affect biological function of the disease, and the impact of promising ncRNA on the function of OS will be further explored. In addition, the biomarkers found in urine or blood by non-invasive procedures is ideal better than collecting tissues. Furthermore, there are great challenges associated with RNA-based therapeutics. On the one hand, RNA drugs can easily approach the targets based on nucleotide hybridization. On the other hand, the delivery method is a challenging process. As a promising new biomarker, ncRNA research is undergoing a rapid change and entering a new area of development. To keep up the pace, better techniques for the detect of new useful ncRNAs need to be implemented, and the evaluation of those potential biomarker should be validated in a larger sample size. In the near future, our team will still be committed to the above studies of OS. Discovering new targets by high throughput sequencing methods, and follow-up studies on pathways are our research goals which can promise the revolution of OS. It is believed that with the further popularization and deepening of research, there will be new thoughts on the early prevention, accurate diagnosis and safer and more effective treatment of OS, and further application of ncRNAs to the diagnosis and treatment of other types of cancer.
